# A Novel Clutter Suppression Method Based on Sparse Bayesian Learning for Airborne Passive Bistatic Radar with Contaminated Reference Signal

**DOI:** 10.3390/s21206736

**Published:** 2021-10-11

**Authors:** Jipeng Wang, Jun Wang, Yun Zhu, Dawei Zhao

**Affiliations:** 1 National Laboratory of Radar Signal Processing, Xidian University, Xi’an 710071, China; jpwang_2@stu.xidian.edu.cn (J.W.); zdw-naruto@163.com (D.Z.); 2 School of Computer Science, Shaanxi Normal University, Xi’an 710062, China; yunzhu@snnu.edu.cn

**Keywords:** airborne passive bistatic radar, multipath signal, clutter suppression, space–time adaptive processing, sparse Bayesian learning

## Abstract

The novel sensing technology airborne passive bistatic radar (PBR) has the problem of being affecting by multipath components in the reference signal. Due to the movement of the receiving platform, different multipath components contain different Doppler frequencies. When the contaminated reference signal is used for space–time adaptive processing (STAP), the power spectrum of the spatial–temporal clutter is broadened. This can cause a series of problems, such as affecting the performance of clutter estimation and suppression, increasing the blind area of target detection, and causing the phenomenon of target self-cancellation. To solve this problem, the authors of this paper propose a novel algorithm based on sparse Bayesian learning (SBL) for direct clutter estimation and multipath clutter suppression. The specific process is as follows. Firstly, the space–time clutter is expressed in the form of covariance matrix vectors. Secondly, the multipath cost is decorrelated in the covariance matrix vectors. Thirdly, the modeling error is reduced by alternating iteration, resulting in a space–time clutter covariance matrix without multipath components. Simulation results showed that this method can effectively estimate and suppress clutter when the reference signal is contaminated.

## 1. Introduction

Having the advantages of both passive bistatic radar (PBR) and airborne radar, airborne passive bistatic radar has aroused extensive research interest from research institutions and scholars as a new type of sensing technology [1,2,3]. As a form of PBR, airborne PBR does not actively transmit a detection signal; instead, it relies on signals transmitted by non-cooperative sources to detect, locate, and track targets. Due to this silent reception mode, airborne PBR has many advantages such as strong battlefield survivability, low cost, and strong anti-stealth ability [4,5,6,7,8]. Additionally, as an airborne radar, its line-of-sight distance increases as its receiving platform rises, which can effectively overcome the long-range shielding problem of traditional ground-based radar due to the curvature and terrain of the earth [9,10]. Additionally, airborne PBR can be quickly deployed because of its strong maneuverability. However, to achieve effective target detection, some of airborne PBR’s problems need to be solved.

In contrast to ground-based radar, due to the movement of the receiving platform, the clutter received by airborne PBR does not concentrate at zero Doppler. Therefore, the ground-based clutter cancellation algorithm is not applicable. Space–time adaptive processing (STAP) is usually used to solve the Doppler-broadened clutter problem in airborne radar [10,11,12]. To date, many STAP methods, such as reduced-dimension (RD) STAP [9,13], reduced-rank (RR) STAP [14,15], sparse recovery (SR) STAP [16,17,18,19], and knowledge-aided (KA) STAP [20], have been proposed. The RR and RD methods can reduce the computational complexity of space–time adaptation. The SR method uses the sparsity of the clutter in spatial–temporal frequency [16]. Through compressed sensing, the SR method can deal with spatial–temporal clutter under the conditions of insufficient, independent, and identically distributed samples to estimate the spatial–temporal clutter spectrum. The KA method uses prior knowledge to improve the accuracy of the estimation of the clutter spectrum.

The abovementioned STAP methods are designed for conventional airborne radar but are also applicable to airborne PBR radar. In recent years, some STAP methods have been proposed for airborne PBR. The authors of [21] designed a sparse Bayes-based STAP and motion parameter estimation method for multistatic airborne RBR that could well-estimate clutter when training samples were insufficient. The authors of [2,22,23,24,25] studied the problem of the high sidelobe of the continuous wave signal commonly used in the external emitter radar, and they designed clutter cancellation algorithms to eliminate the impact of random range sidelobe.

Airborne PBR systems, like traditional airborne radars, use monitoring antennas to receive echo signals from their monitoring areas. However, since airborne PBR does not have a local reference signal like active radar, a separate reference antenna pointing to the non-cooperative transmit source is also required. The reference signal received by the reference antenna and the echo signal are used for clutter cancellation and matched filtering. However, in practical applications, because the civil transmit source used by PBR radar is often in a complex environment, when the reference antenna is used to receive the reference signal, it also receives other scattering points in the environment (such as buildings, trees, and mountains) that refract multipath signals, thus causing the received reference signal to become a contaminated reference signal.

Multipath reference signals cause a series of problems due to the clutter cancellation and target detection of airborne PBR. First of all, multipath clutter in the reference signal matched with the spatial–temporal clutter in the echo signal expand the clutter power spectrum. When the target echoes are close to the multipath clutter region, STAP suppresses the target echoes and the target self-cancellation phenomenon occurs. As a consequence, the performance of STAP is seriously degraded, and when the multipath and target Doppler are close, the target self-cancellation phenomenon occurs; at the same time, when the multipath component in the reference signal matches the target component in the echo signal, a false target situation occurs. Therefore, it is necessary to design an effective algorithm that allows airborne PBR to effectively suppress spatial–temporal clutter and detect targets when the reference signal is contaminated. The blind equalization algorithm in [26,27] was proposed to suppress multipath components in reference signals, but it requires the signal to satisfy the premise of the cyclostationary characteristic. The authors of [28] proposed a sparse l1-rls-based cancellation algorithm to cascade the multipath and main clutter, but the regularization parameter is highly sensitive to environmental changes and is complex in the practice geographical environments, especially in the case of non-uniform clutter. Additionally, the sparse l1-rls-based cancellation method uses Doppler restriction to distinguish multipath clutter, which is likely to cause the false cancellation of the target.

Aiming to solve the contaminated reference signal problem of airborne PBR, the authors of this paper propose a clutter suppression method based on the sparse Bayesian learning (SBL) methods [29]. Firstly, a spatial–temporal clutter snapshot with multipath is expressed in the form of covariance, and then the multipath components are decorrelated in the covariance vector. Finally, the cost function is established, the modeling error is eliminated by EM algorithm, and the direct path spatial–temporal clutter covariance matrix without multipath components is iteratively obtained to suppress multipath clutter. Simulation results showed that the proposed algorithm can effectively estimate and suppress clutter when the reference signal is contaminated. The simulation results also showed that the proposed algorithm could achieve better performance than existing algorithms in different scenarios and simulation conditions.

The remainder of this paper is organized as follows. Section 2 introduces existing problems in detail. Section 3 provides the theoretical analysis and detailed implementation steps of the proposed algorithm. Section 4 validates the performance of the proposed algorithm using simulation results. Conclusions are provided in Section 5.

## 2. Signal and System Model

**Figure 1:**
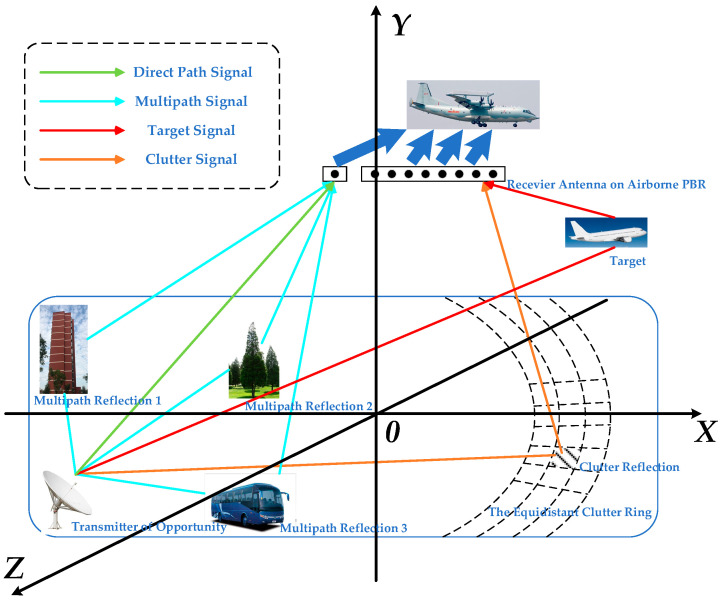
A typical airborne PBR detection system.

In this section, we use formulas to analyze the impact of a contaminated reference signal. The working status of a typical airborne PBR detection system can be expressed as shown in Figure 1.

The reference antenna receives not only a direct wave signal from a non-cooperative emission source but also the multipath signal reflected by the strong point. Therefore, the signal received by the reference channel can be expressed as:

(1) $$\begin{array}{cc} S_{ref}(t) & =C_{d}\sum_{m=0}^{M-1}s\left(t-\tau_{d}-mT_{r}\right)e^{j2\pi f_{d}t}\\ & +\sum_{p=1}^{N_{p}}C_{p}\sum_{m=0}^{M-1}s\left(t-\tau_{p}-mT_{r}\right)e^{j2\pi f_{p}t}+n_{ref}\left(t\right)\\ & =S_{d}\left(t\right)+\sum_{p=1}^{N_{p}}S_{p}\left(t\right)+n_{ref}\left(t\right) \end{array}$$

where M denotes the number of equivalent pulses, $T_{r}$ denotes the pulse repetition interval, $C_{d}$ denotes the complex amplitude of the direct wave signal, $\tau_{d}$ denotes the time delay of a direct wave signal, $f_{d}$ denotes the Doppler frequency of reference direct wave signal, $C_{d}$ denotes the complex amplitude of the p-th multipath signal, $\tau_{p}$ denotes the delay of the p-th multipath signal, $f_{p}$ represents the Doppler frequency of the p-th multipath signal, $S_{d}\left(t\right)$ denotes the complex envelope of the reference signal, $S_{p}\left(t\right)$ represents the complex envelope of the p-th multipath signal, and $n_{ref}\left(t\right)$ denotes the noise of the reference channel.

The signal model received by the echo channel can be expressed as:

(2) $$\left\{\begin{array}{c} H_{0}:S_{echo}\left(t\right)=S_{c}\left(t\right)+n_{\mathrm{ech}o}\left(t\right)\\ H_{1}:S_{echo}\left(t\right)=S_{c}\left(t\right)+S_{tar}\left(t\right)+n_{\mathrm{ech}o}\left(t\right) \end{array}\right.$$

where the $H_{0}$ state indicates that the current distance unit does not contain a target, the $H_{1}$ state indicates that the current distance unit contains a target, $S_{c}\left(t\right)$ denotes the signal of spatial–temporal clutters, $S_{tar}\left(t\right)$ denotes the signal of targets, and $n_{\mathrm{ech}o}\left(t\right)$ denotes the noise of the echo channel.

The components of an airborne PBR system, as analyzed below, receive the reflection model signal from the *i*-th point, and the model can be expressed as:

(3) $$S(n,i,t)=\alpha_{l,i}\sum_{m=0}^{M-1}s_{m}\left(t-\tau_{i}-mT_{r}\right)e^{j2\pi f_{i}t}e^{j2\pi n\vartheta_{i}}$$

where $\alpha_{l,i}$ denotes the complex amplitude of the echo signal from corresponding reflection and $f_{i}$ and $\vartheta_{i}$ denote the temporal and spatial frequency, respectively.

Therefore, the spatial–temporal clutter echo at the 1st distance unit, that is $S_{c}(n,l,t)$, can be expressed as:

(4) $$S_{c}(n,l,t)=\sum_{i=1}^{N_{c}}A_{i}\sum_{m=0}^{M-1}s\left(t-\tau_{l}-mT_{r}\right)e^{j2\pi f_{i}t}e^{j2\pi n\vartheta_{i}}$$

where $A_{i}$ denotes the complex amplitude of the echo signal from corresponding clutter.

The target echo signal in the *q*-th range unit can be expressed as:

(5) $$S_{tar}(n,q,t)=A_{t}\sum_{m=0}^{M-1}s_{m}\left(t-\tau_{q}-mT_{r}\right)e^{j2\pi f_{q}t}e^{j2\pi n\vartheta_{q}}$$

where $A_{t}$ denotes the complex amplitude of the echo signal from the corresponding target.

In actual space–time adaptive processing, the distance unit signal near the distance unit to be detected is generally used as the training sample of clutter to determine whether there is a target in the unit. Therefore, by using the echo signal in the H0 state and an impure reference signal for matched filtering, the following can be obtained:

(6) $$\begin{array}{l} \chi_{C}\left(n,l,t\right)=\int S_{c}\left(n,l,\xi \right)S_{ref}^{*}\left(\xi -t\right)d\xi \\ =\sum_{i=1}^{N_{c}}\varepsilon_{i}e^{j2\pi \left(n-1\right)\vartheta_{i}}\sum_{m=0}^{M-1}e^{j2\pi mT_{r}\left(f_{i}-f_{d}\right)}r_{m}\left(t-\left(\tau_{i}-\tau_{d}\right)-mT_{r}\right)\\ +\sum_{p=1}^{N_{p}}\sum_{i=1}^{N_{c}}\varepsilon_{i,p}e^{j2\pi \left(n-1\right)\vartheta_{i}}\sum_{m=0}^{M-1}e^{j2\pi mT_{r}\left(f_{i}-f_{p}\right)}r_{m}\left(t-\left(\tau_{i}-\tau_{p}\right)-mT_{r}\right)+\chi_{noise}\left(t\right)\\ =\chi_{dc}\left(n,l,t\right)+\sum_{p=1}^{N_{T}}\chi_{pc}\left(n,l,t\right)+\chi_{noise}\left(t\right) \end{array}$$

Therefore, the clutter component of the *l*-th distance unit to be detected is expressed as (for convenience of expression, the range unit serial number ‘*l*’ is omitted later):

(7) $$\chi_{c,l}=\chi_{dc,l}+\sum_{p=1}^{N_{T}}\chi_{pc,l}+\chi_{n,l}$$

In space–time adaptive processing, it is necessary to obtain the clutter covariance matrix of the current distance unit, which cannot be known in advance in practical processing. Generally, the clutter covariance matrix can be estimated from L distance units near the distance unit to be detected; the process can be expressed as:

(8) $$R_{C}=E\left(\chi_{C}\chi_{C}{}^{T}\right)=\frac{1}{L}\sum \chi_{C}\chi_{C}{}^{T}=R_{c_{d}}+\sum_{p=1}^{N_{p}}R_{c_{p}}+R_{n}$$

where ${\left(•\right)}^{T}$ denotes transpose operation.

Then, the weight of space–time adaptive cancellation is obtained by using the clutter covariance matrix:

(9) $$w=\frac{R_{C}^{-1}v\left(f_{s},f_{t}\right)}{v^{H}\left(f_{s},f_{t}\right)R_{C}^{-1}v\left(f_{s},f_{t}\right)}$$

It can be seen that the obtained adaptive clutter cancellation weight w contains multipath components in the covariance RC matrix, which affects clutter cancellation performance. At the same time, the weight mistakenly eliminates the target with the same Doppler as the multipath cost. Therefore, it is necessary to propose corresponding algorithms to suppress the influence of the multipath reference signal.

## 3. Proposed Algorithm

This section explains the proposed algorithm through five subsections. Section 3.1 introduces the representation method of the direct path clutter component under the discretized space–time steering vector. Section 3.2 introduces the representation method of the covariance matrix vector containing multipath clutter under the space–time base, as well as the method of using a decorrelation matrix to suppress the component of the multipath component in the covariance vector. Section 3.3 proposes an algorithm based on sparse Bayesian algorithm to accurately recover the pure direct path clutter covariance. The algorithm reduces the modeling error with the EM iterative method. Section 3.4 provides the estimation method of noise power that needs to be known for the steps discussed in Section 3.2 and Section 3.3. Since the proposed algorithm has a large number of derivation formulas, Section 3.5 skips the intermediate derivation process and provides the primary specific steps for the realization of this algorithm.

### 3.1. Direct Path Clutter Sparse Model

Before analyzing the multipath component, this subsection first proposes the observation model of direct path clutter. As in traditional airborne radar, the clutter of a certain range unit received by the array can be expressed as:

(10) $$X_{dc}=\sum_{i=1}^{Nc}\alpha_{i}s\left(f_{s,i},f_{d,i}\right)+n_{c}$$

(11) $$s\left(f_{s},f_{d}\right)=s_{s}\left(f_{s}\right)⊗s_{d}\left(f_{d}\right)$$

where $n_{c}$ denotes the thermal noise received by PBR; $\alpha_{i}$ denotes the complex amplitude of reflection point; $s\left(f_{s,i},f_{d,i}\right)$ denotes the spatial–temporal vector of the ground reflection point; and $s_{s}\left(f_{s}\right)$ and $s_{d}\left(f_{d}\right)$ represent temporal steering vector and spatial steering vector, respectively, which can be expressed as:

(12) $$s_{s}\left(f_{s}\right)=\left[1,\exp\left(j2\pi f_{s}\right),\cdots ,\exp\left(j2\pi (N-1)f_{s}\right)\right]$$

(13) $$s_{d}\left(f_{d}\right)=\left[1,\exp\left(j2\pi f_{d}\right),\cdots ,\exp\left(j2\pi (M-1)f_{d}\right)\right]$$

In conventional SR space–time adaptive processing algorithms, spatial–temporal clutter is usually estimated by constructing an over-complete sparse recovery dictionary. The general spatial–temporal guidance dictionary is obtained by discretizing the angle Doppler plane. The entire normalized space plane uniformly extends the space frequency axis and the Doppler axis into $T_{s}\times T_{r}$ grid points in the field, where $T_{s}=\eta_{s}N$, $T_{r}=\eta_{r}N$, and $\eta_{s},\eta_{r}>1$; the discretized spatial frequency and Doppler frequency interval are expressed as $\Delta f_{s}=\frac{1}{T_{s}}$ and $\Delta f_{d}=\frac{1}{T_{r}}$, respectively; and the discrete grid points correspond to the guidance vector in the spatial–temporal guidance dictionary. Assuming that all clutter points are just located on the grid points, the direct clutter in Equation (10) can be expressed as [16]:

(14) $$X_{dc}=\Phi \alpha +n_{c}$$

where Φ represents the constructed sparse recovery dictionary (its dimension is $NM\times T_{s}T_{r}$), α is the vector of sparse recovery complex coefficients, and Φ can be expressed in detail as follows [16]:

(15) $$\Phi =\left[s\left(f_{s,1},f_{d,1}\right),s\left(f_{s,2},f_{d,2}\right),\ldots ,s\left(f_{s,T_{s}},f_{d,T_{r}}\right)\right]$$

### 3.2. Covariance Matrix with Multipath Clutter and Its Decorrelation

According to the analysis in Section 2, the components of multipath clutter are consistent with those of main clutter. The only difference between multipath clutter and direct clutter is the addition of a complex factor $\rho_{n}$; therefore, the clutter observation model with main and multipath clutter can be obtained as:

(16) $$X_{c}=X_{dc}+X_{pc}+n=\left(1+\sum_{n=1}^{Np}\rho_{n}\right)\sum_{i=1}^{Nc}\alpha_{i}s\left(f_{s,i},f_{d,i}\right)+n_{c}$$

This can be deformed to obtain:

(17) $${\hat{X}}_{c}=\frac{X_{c}}{\left(1+\sum_{n=1}^{Np}\rho_{n}\right)}=\sum_{i=1}^{Nc}\alpha_{i}s\left(f_{s,i},f_{d,i}\right)+{\hat{n}}_{c}=\Phi \alpha +{\hat{n}}_{c}$$

where ${\hat{X}}_{c}$ denotes the spatial–temporal snapshot signal with a multipath component and ${\hat{n}}_{c}$ denotes the noise error after deformation. Since the number of multipaths $N_{P}$ and coefficients $\rho_{n}$ are unknown, the recovery coefficient cannot be directly calculated in the sparse model. This chapter uses the covariance matrix to solve this problem.

Given T snapshots, the covariance matrix of the spatial–temporal clutter of the current range unit can be expressed as:

(18) $${\hat{R}}_{c}=\frac{1}{T}\sum_{t=1}^{T}{\hat{X}}_{c}{\hat{X}}_{c}{}^{H}=\Phi \left(\frac{1}{T}\sum_{t=1}^{T}\alpha \alpha^{H}\right)\Phi^{H}+\sigma^{2}I_{c}$$

where $\sigma^{2}$ denotes noise variance and $I_{c}$ is the identity matrix of $T_{s}T\times_{r}T_{s}T_{r}$ dimensions.

According to formulas (16)–(18), the covariance moment ${\hat{R}}_{c}$ of clutter component ${\hat{X}}_{c}$ also contains a clutter multipath component. Therefore, it is necessary to first decorrelate the matrix ${\hat{R}}_{c}$ and then use the decorrelated ${\hat{R}}_{c}$ to solve the sparse model so as to obtain the value of the coefficient vector α.

The m-th column vector ${\hat{r}}_{m}$ of matrix ${\hat{R}}_{c}$ can be expressed as:

(19) $${\hat{r}}_{m}=\left({\hat{R}}_{c}-\sigma^{2}I\right)e_{m}=\Phi u_{m}+\xi_{m}$$

where $u_{m}$ denotes the complex sparse vector of ${\hat{r}}_{m}$ under the base Φ and $\xi_{m}$ is the corresponding error.

Due to the presence of multipath clutter components, the elements in the matrix ${\hat{R}}_{c}$ are not only distributed on the diagonal. Therefore, it is necessary to first decorrelate the matrix ${\hat{R}}_{c}$ and the error vector $\xi_{m}$ using the matrix $Q_{m}$, and then the covariance vector after the decorrelation can be expressed in the spatial–temporal overcomplete basis Φ as:

(20) $${\bar{\hat{r}}}_{m}=Q_{m}^{-1/2}{\hat{r}}_{m}=Q_{m}^{-1/2}\Phi {\tilde{u}}_{m}+Q_{m}^{-1/2}\xi_{m},\;m=1,\cdots ,M$$

where the decorrelation matrix $Q_{m}$ is expressed as:

(21) $$Q_{m}=E\left(\xi_{m}\xi_{m}{}^{H}\right)=\frac{1}{T}{\hat{R}}_{c}{}_{\left(m,m\right)}{\hat{R}}_{c}$$

The estimation error $Q_{m}^{-1/2}\xi_{m}$ of the covariance vector ${\bar{\hat{r}}}_{m}$ is independent and identically distributed. After decorrelation, the covariance of the error vector is the unit diagonal matrix, which satisfies the following relationship:

(22) $$E\left\{\left(Q_{m}^{-1/2}\xi_{m}\right){\left(Q_{m}^{-1/2}\xi_{m}\right)}^{H}\right\}=I_{M\times M}$$

By substituting the decorrelation matrix $Q_{m}=\frac{1}{T}R_{m,m}R$ into Equation (20), the following relationship is obtained:

(23) $$\begin{array}{l} {\bar{\hat{r}}}_{m}=Q_{m}^{-1/2}{\hat{r}}_{m}\\ \;=\sqrt{T}{\hat{R}}_{c}{}^{-1/2}\Phi \sum_{i=1}^{M}f_{mi}{\tilde{u}}_{m}+Q_{m}^{-1/2}\xi_{m},\;m=1,\cdots ,M \end{array}$$

where $f_{mi}$ is the element in the m-th row and the i-th column of $R_{c}{}^{-T/2}$.

In Equation (20), ${\tilde{u}}_{m}$ (m=1,⋯,M) always has the same spatial–temporal sparsity, so the signal covariance vector $\bar{\hat{r}}$ (m=1,⋯,M) can be linearly expressed by the same basis function in the spatial–temporal domain overcomplete basis $\sqrt{T}R^{-1/2}\Phi$.

Make $\tilde{\Phi }=\sqrt{T}R_{c}{}^{-1/2}\Phi$, ${\bar{\tilde{u}}}_{m}=\sum_{i=1}^{M}f_{mi}{\tilde{u}}_{m}$, ${\bar{\xi }}_{m}=Q_{m}^{-1/2}\xi_{m}$; then, Equation (23) is transformed into the following form:

(24) $${\bar{\hat{r}}}_{m}=\tilde{\Phi }{\bar{\tilde{u}}}_{m}+{\bar{\xi }}_{m},\;m=1,\cdots ,M$$

### 3.3. Proposed Algorithm Based on Sparse Bayesian Learning

In the previous subsections, the spatial–temporal clutter covariance sparse model was derived. In this subsection, the covariance of only pure direct clutter is reconstructed by sparse Bayesian learning.

The covariance vector estimation error ${\bar{\xi }}_{m}$ follows the complex Gaussian distribution with a mean value of zero, and the covariance is $I_{M}$, that is [29]:

(25) $${\bar{\xi }}_{m}∼\mathrm{CN}\left(0,I_{M}\right)$$

Suppose that ${\bar{\tilde{u}}}_{m}$ follows the complex Gaussian distribution of zero mean, that is:

(26) $${\bar{\tilde{u}}}_{m}∼\mathrm{CN}\left(0,\Gamma \right)$$

where $\Gamma =\mathrm{diag}\left(\gamma_{1},\cdots ,\gamma_{N}\right)$, $\gamma =\left[\gamma_{1},\cdots ,\gamma_{N}\right]$, in which γ represents the power distribution of direct clutter in a spatial–temporal over-complete basis $\tilde{\Phi }$.

The conditional probability density function of the spatial–temporal covariance ${\bar{R}}_{c}$ of the direct wave clutter to be estimated with respect to $\bar{U}$ can be expressed as:

(27) $$p\left({\bar{R}}_{c}\left|\bar{U}\right.\right)={\left|\pi I_{M}\right|}^{-1}\exp\left\{-{\left({\bar{R}}_{c}-\tilde{\Phi }\bar{U}\right)}^{H}\left({\bar{R}}_{c}-\tilde{\Phi }\bar{U}\right)\right\}$$

where $\bar{U}=[{\bar{\tilde{u}}}_{1};{\bar{\tilde{u}}}_{2};\ldots ;{\bar{\tilde{u}}}_{m}]$.

The posterior probability density function of $\bar{U}$ can be expressed as:

(28) $$p\left(\bar{U}\left|\gamma \right.\right)={\left|\pi \Sigma_{\bar{U}}\right|}^{-1}\exp\left\{-\left[{\left(\bar{U}-\mu_{\bar{U}}\right)}^{H}\Sigma_{\bar{U}}{}^{-1}\left(\bar{U}-\mu_{\bar{U}}\right)\right]\right\}$$

where $\mu_{\bar{U}}$ and $\Sigma_{\bar{U}}$ denote the first and second moments of $\bar{U}$, respectively [29]:

(29) $$\mu_{\bar{U}}=\Gamma {\tilde{\Phi }}^{H}{\left(I_{M}+\tilde{\Phi }\Gamma {\tilde{\Phi }}^{H}\right)}^{-1}{\bar{R}}_{c}$$

(30) $$\Sigma_{\bar{U}}=\Gamma -\Gamma {\tilde{\Phi }}^{H}{\left(I_{M}+\tilde{\Phi }\Gamma {\tilde{\Phi }}^{H}\right)}^{-1}\tilde{\Phi }\Gamma$$

The likelihood function of ${\bar{R}}_{c}$ with respect to γ can be expressed as:

(31) $$\begin{array}{l} p\left({\bar{R}}_{c}\left|\gamma \right.\right)=\int p\left({\bar{R}}_{}\left|\bar{U}\right.\right)p\left(\bar{U}\left|\gamma \right.\right)d\bar{U}\\ \;={\left|\pi \Sigma_{{\bar{R}}_{c}}\right|}^{-1}\exp\left\{-{\bar{R}}_{c}{}^{H}\Sigma_{\bar{R}}{\bar{R}}_{c}\right\} \end{array}$$

where:

(32) $$\Sigma_{{\bar{R}}_{c}}=I_{M}+\tilde{\Phi }\Gamma {\tilde{\Phi }}^{H}$$

The estimation of γ can be obtained by setting the likelihood function. Next, the γ is updated step by step through the EM algorithm.

The objective function is constructed according to the maximum likelihood criterion, and the quantity related to the γ is removed to obtain the following objective function:

(33) $$L\left(\gamma \right)=\ln\left[p\left({\bar{R}}_{c}\left|\gamma \right.\right)\right]\propto \ln\left|\Sigma_{{\bar{R}}_{c}}\right|+{\bar{R}}_{c}{}^{H}\Sigma_{{\bar{R}}_{c}}{}^{-1}{\bar{R}}_{c}$$

Find the partial derivative about $\gamma_{n}$ on both sides of the equation above and make the partial derivative zero, that is:

(34) $$\frac{\partial L\left(\gamma \right)}{\partial \gamma_{n}}=M{\left(\gamma_{n}\right)}^{-1}-{\left\|{\bar{R}}_{c}\right\|}_{2}^{2}{\left(\gamma_{n}\right)}^{-2}=0,n=1,\cdots ,N$$

In iteration *q* + 1, $\gamma_{n}$ can be updated by the following equation:

(35) $$\gamma_{n}{}^{\left(q+1\right)}={\left\|{\left(\mu_{\bar{U}}{}^{\left(q\right)}\right)}_{n\cdot }\right\|}_{2}^{2}/M+{\left(\Sigma_{\bar{U}}{}^{\left(q\right)}\right)}_{n,n},n=1,\cdots ,N$$

where ${\left(\mu_{\bar{U}}{}^{\left(q\right)}\right)}_{n\cdot }$ denotes the n-th row vector of $\mu_{\bar{U}}{}^{\left(q\right)}$ and ${\left(\Sigma_{\bar{U}}{}^{\left(q\right)}\right)}_{n,n}$ denotes the n-th element in the n-th row of $\Sigma_{\bar{U}}{}^{\left(q\right)}$.

According to Equation (35), when the algorithm iteration is close to convergence, a large number of elements in γ are 0. At this time, errors easily occur in numerical calculation. In order to avoid these numerical abnormalities, the following formula can be used to replace the update of $\gamma_{n}$ in the above formula:

(36) $$\gamma_{n}{}^{\left(q+1\right)}=\frac{1}{M}{\left\|{\left(\mu_{\bar{U}}{}^{\left(q\right)}\right)}_{n\cdot }\right\|}_{2}^{2}/\left[1-{\left(\Sigma_{\bar{U}}{}^{\left(q\right)}\right)}_{n,n}/\gamma_{n}{}^{\left(q\right)}\right]+\zeta ,\;n=1,\cdots ,N$$

where ζ is a constant and its function prevents a large number of zero elements from being produced in γ, which is usually taken as a very small positive number (such as $\zeta =10^{-4}$).

The calculated γ represents the distribution of direct clutter on the spatial–temporal over-complete $\tilde{\Phi }$, and the spatial–temporal covariance matrix $R_{c}$ containing only direct clutter can be obtained by γ and $\tilde{\Phi }$ reconstruction.

### 3.4. Noise Power Estimation

To set the spatial–temporal clutter covariance vector, one needs to know the variance $\sigma^{2}$ of receiver noise in advance, that is, the power of noise. The estimated value of noise variance can be obtained by averaging the small eigenvalues after eigendecomposition of spatial–temporal clutter covariance matrix ${\hat{R}}_{c}$, that is:

(37) $${\hat{\sigma }}^{2}=\frac{1}{M-K}\sum_{m=K+1}^{M}\lambda_{m}$$

where $\lambda_{m}$ denotes the m-th large eigenvalue after the eigenvalue decomposition of spatial–temporal clutter covariance matrix ${\hat{R}}_{c}$ and K is the maximum spatial–temporal clutter degree of freedom that is usually set to NM-1.

### 3.5. Algorithm Summary

The algorithms proposed in this section are summarized in Table 1.

## 4. Simulations and Performance Analyses

In this section, five sets of simulation experiments are used to verify the effectiveness of the algorithm proposed in this paper and its advantages compared to existing algorithms. The experiments in Section 4.1 and Section 4.2 prove the improvement of the proposed algorithm compared to the algorithm through the two most commonly used verification experiments in space–time adaptive processing algorithm, namely the space–time clutter spectrum and the of signal-to-noise ratio in improvement factor. The Monte Carlo experiments in Section 4.3 and Section 4.4 set more comprehensive conditions and quantitatively analyzed the advantages of the proposed algorithm in clutter cancellation performance compared to the existing algorithms. Section 4.5 further verifies the advantages of the algorithm in this paper by comparing the proposed algorithm with the existing algorithm and comparing the target detection results of different algorithms.

### 4.1. Spatial–Temporal Clutter Spectrum

This experiment evaluated the performance of the proposed algorithm by comparing the spatial–temporal clutter spectra obtained by the proposed algorithm and three other existing algorithms: the Full-STAP, Sparse Recovery (SR-STAP), and cascade cancellation STAP (CM-STAP) algorithms. Among them, the Full-STAP algorithm is representative of the conventional STAP algorithm or its dimensionality reduction and rank reduction methods; and the SR-STAP algorithm is representative of the sparse STAP algorithm; the first step of the cascade cancellation of the STAP algorithm uses the RLS algorithm, and the second step uses the Full-STAP algorithm. In addition, for fair comparison performance, the SR method, the CM-STAP method, and the method proposed by this paper all divide the spatial–temporal grid into 60*60. The settings of other major parameters are shown in Table 2.

**Figure 2:**
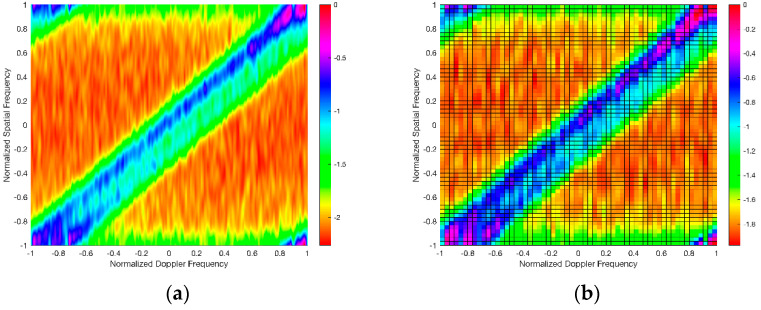

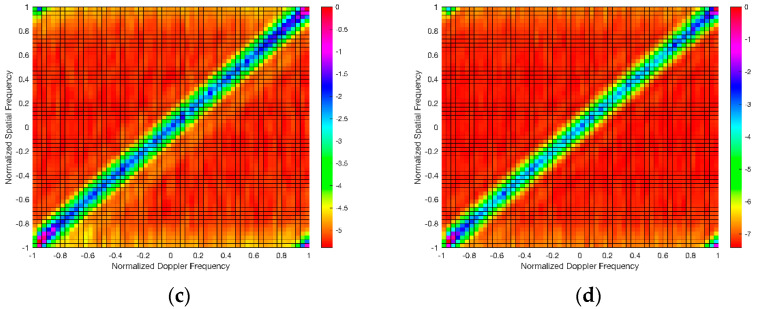
Spatial–temporal clutter spectra in scene 1 using the (**a**) Full–STAP, (**b**) SR–STAP, (**c**) CM–STAP, and (**d**) proposed algorithms.

**Figure 3:**
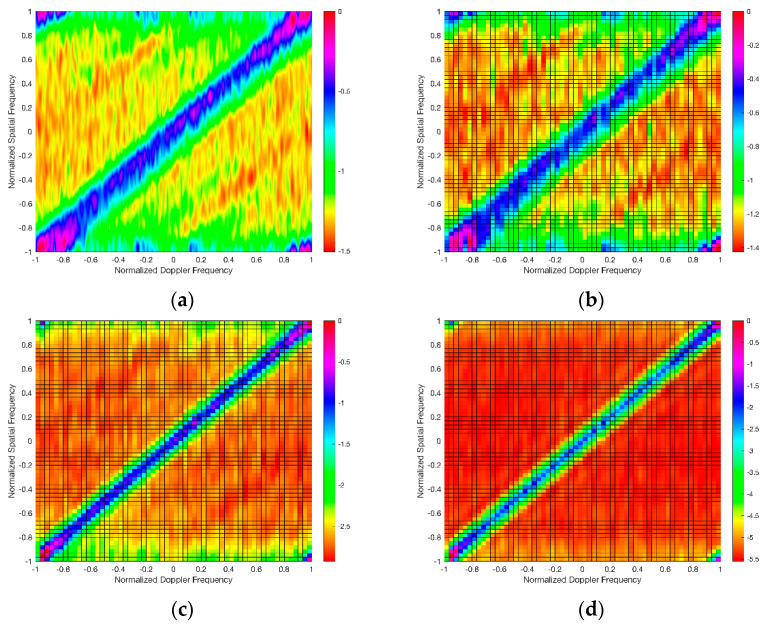
Spatial–temporal clutter spectra in scene 2 using the (**a**) Full–STAP, (**b**) SR–STAP, (**c**) CM–STAP, and (**d**) proposed algorithms.

At the same time, in order to show that the advantages of the algorithm proposed in this article are more obvious, the experimental environment was divided into scenes 1 and 2 according to the state of the reference multipath signal; scene 1 represents an environment with a small number of multipaths in the reference signal and a high intensity, and scene 2 represents an environment in which the multipath in the reference signal is caused by dense and strong reflection points, with a large number and a lower intensity than those of scene 1. The four abovementioned algorithms were used to process scenes 1 and 2 to obtain the spatial–temporal clutter spectra shown in Figure 2 and Figure 3.

Figure 2 and Figure 3 show the spatial–temporal clutter responses of scenes 1 and 2, respectively, with the processing of four algorithms. It can be seen from Figure 2a,b and Figure 3a,b that in the state of contaminated reference signal, the spatial–temporal clutter spectra of the Full-STAP algorithm and the SR-STAP method were expanded to increase the error and detection blind area of clutter cancellation. It can be seen from Figure 2c,d and Figure 3c,d that the cascade cancellation algorithm and the method in this paper could achieve effective multipath clutter suppression.

Since the first step of the cascade cancellation algorithm uses the RLS adaptive cancellation algorithm to perform iterative operations, a certain number of training samples are required to make the RLS algorithm enter a steady state such that when the number of multipaths increases and the intensity divisions are denser, it reduces convergence speed and affects suppression performance; however, since the proposed algorithm uses decorrelation in the clutter covariance matrix, it is not affected by the number of multipaths. Therefore, in scene 2 (see Figure 3c,d), the proposed algorithm achieved better multipath clutter suppression performance than the cascaded destructive STAP algorithm.

### 4.2. Improvement Factor of Signal-to-Noise Ratio

In order to further verify the multipath clutter suppression performance of the proposed method, this experiment simulated the relationship between the improvement factor (IF) of the signal-to-noise ratio and the normalized Doppler frequency of the target. IF can be obtained via Equation (38):

(38) $$IF=\frac{\sigma_{n}^{2}}{NM}\frac{{\left|w^{H}s\right|}^{2}}{w^{H}R_{c}w}$$

where w denotes the estimated STAP weight vector in Equation (9).

**Figure 4:**
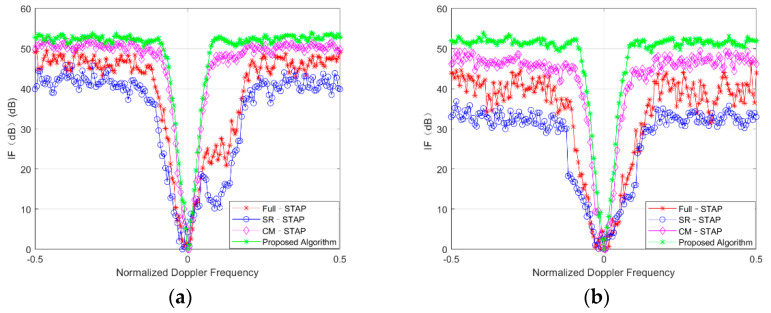
Comparison of improvement factor (IF) with different algorithms in (**a**) scene 1 and (**b**) scene 2.

Figure 4a,b shows the SINR loss curves of environments 1 and 2, respectively, processed by the four algorithms. It can be seen that in environments 1 and 2, both the Full-STAP and SR-STAP algorithms produced deep zero limits where multipath clutter existed, which indicates that targets with these zero limits are eliminated in error when using these two algorithms; the CM-STAP and proposed algorithms, however, could effectively eliminate multipath clutter in advance, which produced a small response at the multipath position during STAP and greatly reduced the phenomenon of target self-cancellation. Figure 4a,b shows that in scenes 1 and 2, the algorithm proposed in this paper has more obvious performance advantages than the CM-STAP algorithm.

### 4.3. Performance with Different Number of Multipaths

In our experimental simulations with different algorithms, the quantitative analysis was accompanied by an increase in the number of multipaths, which affected the clutter suppression performance of the algorithm. The number of multipaths varied from 0 to 80. For the quantity value of each multipath, the SINR loss value was recorded as the average value of 200 iterations.

**Figure 5:**
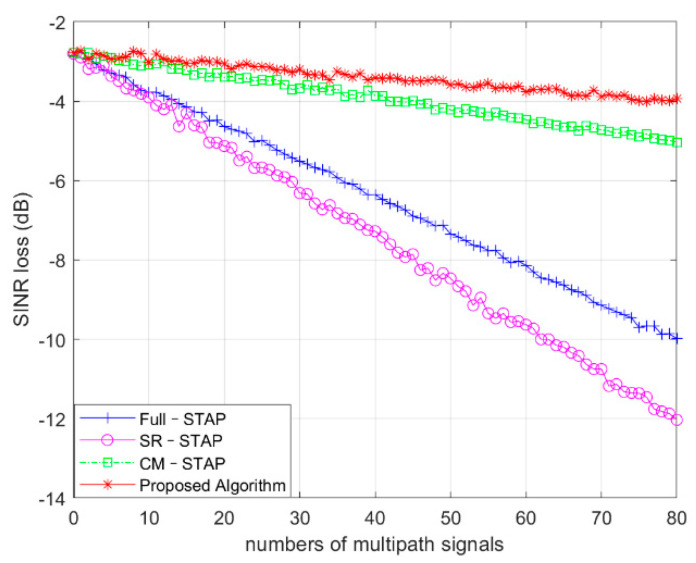
Performance with different numbers of multipaths.

As shown in Figure 5, as the number of multipaths of the Full-STAP and SR-STAP algorithms increased, the number of multipaths increased, the loss of clutter suppression SINR significantly decreased, and the clutter suppression performance gradually deteriorated. The CM-STAP and proposed algorithms were less affected by the number of multipaths, and the proposed algorithm showed better clutter suppression performance than the CM-STAP algorithm when the number of multipaths increased.

### 4.4. Performance with Different Multipath Intensity

In this experiment simulation with different algorithms, we quantitatively analyzed the influence of the algorithm’s clutter suppression performance with the increase of multipath intensity. The number of multipaths was fixed, and the intensity of the multipath changed from –10 to –50 dB; the SINR loss value corresponding to each multipath strength value was the average result of 200 experiments.

**Figure 6:**
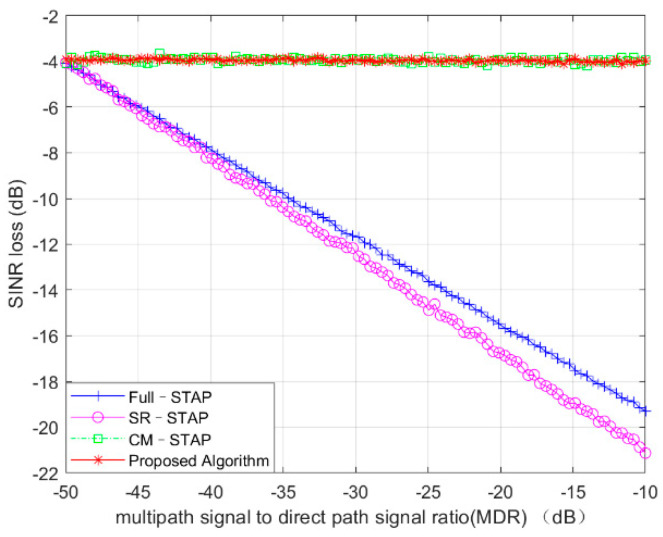
Performance with different multipath intensities.

As shown in Figure 6, the simulation results show that with the increase of multipath intensity, the SINR loss performance of the Full-STAP and SR-STAP algorithms significantly increased with multipath clutter and the clutter suppression performance worsened. However, the CM-STAP and proposed algorithms were not affected by clutter intensity, and their performance was relatively stable.

### 4.5. Performance of Distance Dimension

In this experiment, the target detection performance in the presence of multipath clutter was verified via simulations. For the convenience of discussion, this part of the simulation was set as scene 1, and the target was set at the Doppler position where multipath clutter existed at the 84th range unit. As such, the results in the range dimension without STAP algorithm and after processing with the abovementioned four algorithms can be compared.

**Figure 7:**
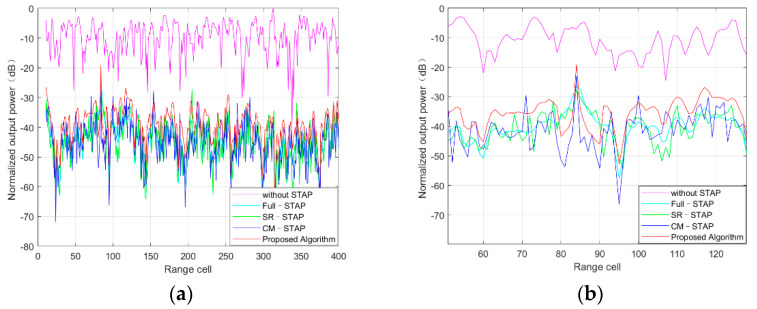
Performance of distance dimension in (**a**) the 0–400th range bin and (**b**) the 60–120th range bin details.

As can be seen from Figure 7a,b when the STAP algorithm was not used, the target could not be detected under clutter; however, the target could be detected using all four methods. The Full-STAP and SR-STAP algorithms could produce deep null at the position where multipath clutter existed, suppress clutter, and weaken target strength, so target detection was poor. Although the CM-STAP algorithm effectively suppressed the multipath clutter in the first step and had better multipath clutter suppression performance, it limited the target detection performance to a certain extent due to the influence of multipath clutter intensity in the RLS algorithm. The algorithm proposed in this paper does not suppress the clutter at the multipath position directly because of its initial clutter decorrelation, so it has better target detection performance at positions where multipath clutter exists.

## 5. Conclusions

Aiming to solve the problem of degraded clutter suppression performance due to the contamination of the reference signal in airborne PBR, a novel algorithm of multipath clutter suppression based on sparse Bayesian learning was proposed in this paper.

Firstly, the spatial–temporal clutter snapshot with multipath is expressed in the form of covariance, and then the multipath components are decorrelated in the covariance vector. Finally, the cost function is established, the modeling error is eliminated by the EM algorithm, and the direct path spatial–temporal clutter covariance matrix without multipath components is iteratively obtained to suppress multipath clutter.

The simulation showed that the proposed algorithm could achieve better performance than existing algorithms regarding the space–time clutter spectrum and the signal-to-noise ratio improvement factor; the algorithm could better maintain stable performance than existing algorithms when the number and strength of multipath increased; and when the target to be detected and the multipath clutter had the same Doppler characteristics, the proposed algorithm showed better target detection performance.

## Figures and Tables

**Table 1:** Summary of algorithm steps.

Summary
(1) Initialization the noise variance ${\hat{\sigma }}^{2}$ and the spatial–temporal clutter spectrum γ.
(2) Construct the clutter covariance vector according to Equation (19).
(3) Decorrelate the clutter covariance vector according to Equation (23).
(4) Update the spatial–temporal clutter spectrum $\gamma_{n}$ according to Equation (29), Equation (30), and Equation (36) to obtain $\gamma_{n}{}^{q+1}$.
(5) Update the noise variance according to Equation (37).
(6) Repeat steps (4) and (5) until the convergence conditions are met to obtain the spatial–temporal clutter spectrum of direct signal clutter
(7) According to Equation (17) and Equation (9), the direct clutter covariance matrix $R_{c}$ is constructed by using the spatial–temporal position, and the spatial–temporal cancellation weight w in Equation (9) is obtained.

**Table 2:** Simulation parameters.

Parameter	Value
Equivalent PRF	1000
Signal bandwidth	8 MHz
Signal wavelength	0.5 m
Main beam look direction	side-looking
Airborne platform velocity	250 m/s
Airborne platform height	2000 m
Array element spacing	0.25 m
Number of antenna elements	16
Number of equivalent pulses	16

## Data Availability

No new data were created or analyzed in this study, so data sharing is not applicable.
